# Medigap Protection and Plan Switching Among Medicare Advantage Enrollees With Cancer

**DOI:** 10.1001/jamahealthforum.2025.2018

**Published:** 2025-06-07

**Authors:** Youngmin Kwon, Shelley A. Jazowski, Xin Hu, Emma M. Achola, John A. Graves, Laura M. Keohane, Stacie B. Dusetzina

**Affiliations:** 1 Department of Health Policy, Vanderbilt University Medical Center, Nashville, Tennessee; 2 Department of Social Sciences and Health Policy, Wake Forest University School of Medicine, Winston-Salem, North Carolina; 3 Department of Radiation Oncology, Emory University School of Medicine, Decatur, Georgia

## Abstract

**Importance:**

An increasing number of Medicare beneficiaries with cancer report Medicare Advantage (MA) coverage, but certain features of MA (eg, utilization management) may impede access to cancer care. MA beneficiaries may desire to switch to traditional Medicare (TM), which imposes little to no utilization restrictions, but switching may be challenging because access to Medigap—providing financial protections against high cost sharing in TM—is limited by medical underwriting of beneficiaries applying after initial Medicare enrollment in most states.

**Objective:**

To examine associations of Medigap guaranteed issue protections that prohibit medical underwriting with MA disenrollment among beneficiaries newly diagnosed with cancer.

**Design, Setting, and Participants:**

This retrospective cohort study examined Medicare beneficiaries 69 years and older who were newly diagnosed with cancer from 2014 to 2019 in the Surveillance, Epidemiology, and End Results Program–linked Medicare database. Beneficiaries continuously enrolled in Medicare Parts A and B for 4 years before to 1 year after diagnosis were included. Data were analyzed from October 2024 to April 2025.

**Exposure:**

A new cancer diagnosis.

**Main Outcomes and Measures:**

The outcome was switching to TM. Among those who were initially enrolled in MA, a difference-in-differences design was implemented to compare changes in the probability of MA disenrollment between beneficiaries diagnosed in 3 guaranteed issue states (New York, Connecticut, and Massachusetts) vs other 13 states contributing to the Surveillance, Epidemiology, and End Results Program registry, before and after diagnosis.

**Results:**

The study included 180 057 MA beneficiaries 69 years and older who were newly diagnosed with cancer (44.5% diagnosed at age 69-75 years; 51.5% male; 8.0% Hispanic; 7.4% non-Hispanic Black; 78.5% non-Hispanic White; and 6.1% another or unknown race and ethnicity). The rate of switching in guaranteed issue states increased from 2.1% to 4.7% following diagnosis but remained unchanged in other states (1.8% to 1.7%), corresponding to a difference-in-differences of 2.5 percentage points (95% CI, 1.9-3.2 percentage points; *P* < .001), or a 120% relative change. This differential increase was concentrated among beneficiaries who were younger, non-Hispanic White, diagnosed with distant-stage or rarer cancers, or enrolled in plans with more generous coverage (eg, PPO plans) or lower plan star ratings.

**Conclusions and Relevance:**

In this cohort study, state Medigap guaranteed issue protections were associated with higher rates of switching to TM among MA beneficiaries newly diagnosed with cancer. These findings underscore the protective association of state Medigap regulations in facilitating a switch to TM (especially among beneficiaries who likely desired more flexibility in accessing and receiving care) and illuminate potential disparities in switching that may reflect unequal abilities to compare and afford plans.

## Key Points

**Question:**

Do patterns of Medicare Advantage (MA) disenrollment differ between MA beneficiaries with cancer in states with and without Medigap guaranteed issue protections?

**Findings:**

In this cohort study of 180 057 MA beneficiaries 69 years and older who were newly diagnosed with cancer from 2014 to 2019, beneficiaries diagnosed in states providing Medigap guaranteed issue protection were 2.5 percentage points more likely to switch to traditional Medicare following diagnosis (a 120% relative increase), compared to beneficiaries in other states.

**Meaning:**

State Medigap regulations may facilitate a switch to traditional Medicare among beneficiaries with cancer who have needs for high-cost specialty care.

## Teaser

This cohort study examines associations of Medigap guaranteed issue protections that prohibit medical underwriting with Medicare Advantage disenrollment among beneficiaries newly diagnosed with cancer.

## Introduction

The rapid growth in Medicare Advantage (MA) enrollment has prompted concerns regarding the quality of care and plan satisfaction within MA.^1,2^ MA plans allow Medicare beneficiaries to receive care via private plans, which emphasize lower cost, care coordination, and supplemental benefits, as opposed to traditional Medicare (TM) in which beneficiaries’ cost sharing may be steeper but there are fewer restrictions in accessing care.^3^ On initial Medicare enrollment, beneficiaries choose from Medicare coverage options in accordance with their preferences, care needs, and financial circumstances.^4^ This choice may be relatively inconsequential for beneficiaries in good health with few care needs, who may prefer MA to attain lower premiums and cost sharing.^5^ In contrast, those diagnosed with serious illnesses may find care management in MA as obstacles to care and may prefer the flexibility in access to care under TM.^6,7^ Due to high cost sharing in TM (eg, 20% coinsurance on Part B services without out-of-pocket maximum), Medicare supplemental coverage may be necessary for care affordability in TM.^8,9^ As a result, most TM beneficiaries (without employer-sponsored retiree benefits or Medicaid supplemental coverage) purchase private supplemental insurance, known as Medigap, which covered more than 42% of all TM beneficiaries in 2022.^10^

However, beneficiaries who initially selected MA at the time of Medicare enrollment may encounter substantial hurdles to obtaining Medigap if they ever contemplate switching to TM.^11,12,13^ Medigap insurers can medically underwrite beneficiaries who apply outside of the one-time, federally guaranteed issue period that spans the first 6 months of Part B enrollment for beneficiaries 65 years and older.^12^ Beyond this federal floor, only 4 states (Connecticut, New York, Massachusetts, and Maine) have established further guaranteed issue protections, requiring Medigap insurers to offer coverage once per year without medical underwriting, along with community rating in the Medigap market.^14^ Thus, in most states, MA beneficiaries with costly preexisting conditions may have difficulty obtaining Medigap coverage. Without Medigap, MA beneficiaries who otherwise would desire a switch to TM may be forced to remain in MA due to high cost sharing in TM.^15,16^

This barrier to obtaining Medigap is salient for beneficiaries with cancer, who develop acute needs for costly care immediately following diagnosis. Such needs may necessitate a switch to TM for improved access to specialty care,^17,18,19^ yet the catastrophic costs of cancer care may leave beneficiaries vulnerable to high switching cost if they are unable to obtain Medigap coverage.^20,21,22^ With anecdotal accounts detailing the obstacles to plan switching among beneficiaries newly diagnosed with cancer,^11,13^ there is a need for a more thorough investigation of plan switching in this population. In this study, we leveraged state-level variation in Medigap guarantee issue protections to examine its association with plan switching among MA beneficiaries newly diagnosed with cancer.

## Methods

### Data Source

The primary data source was the Surveillance, Epidemiology, and End Results (SEER) Program–linked Medicare data, which captures nearly all incident cancer cases among participating state cancer registries and provides linked Medicare data for eligible patients with cancer using a deterministic algorithm (>96% of eligible patients are linked).^23^ Data for this study encompassed cases for 14 common cancer sites, accounting for nearly 81% of all incident cancer cases in the US (see the eMethods in Supplement 1 for more details).^24^

The Vanderbilt University Medical Center institutional review board deemed this study exempt from review and that informed consent was not needed because SEER-Medicare data are deidentified. We followed the Strengthening the Reporting of Observational Studies in Epidemiology (STROBE) reporting guidelines.

### Study Population

Using the 2010-2020 SEER and linked Medicare enrollment files, we constructed a beneficiary-year panel of enrollment data for beneficiaries 69 years and older who were newly diagnosed with cancer from 2014 to 2019. We included yearly enrollment records for each beneficiary from the fourth year prior to the diagnosis (T_−4_) and 1 year after diagnosis (T_1_). Among those continuously enrolled in MA for the entire enrollment duration at 4 years before diagnosis (T_−4_), we followed their Medicare plan selection (staying in MA or switching to TM) in each subsequent year from T_−3_ (ie, the first year in which we can observe switching) until the point of MA disenrollment or through T_1_. We excluded beneficiaries who died in the year of diagnosis (for whom we do not observe plan selection in T_1_), were dually enrolled in Medicaid (which covers TM cost sharing^25^), were eligible for Medicare due to disability or end-stage kidney disease (who are exempt from existing state Medigap protections^26^), and were not continuously enrolled in Parts A and B for the entire enrollment window when they were alive (eFigure 1 in Supplement 1).

### Exposure and Outcome

We identified beneficiaries who were diagnosed in 3 states—Connecticut, Massachusetts, and New York—with guaranteed issue protections that are represented in SEER.^14^ The outcome was a binary indicator, which is 1 for the beneficiary-year when a switching to TM occurred. For simplicity, we considered plan switching only once, censoring observation at the point of switching to TM. We categorized plan selection based on the enrollment information for December in each year. For those who died in T_1_, we used their enrollment information in the month of death to categorize coverage for the death year.

### Covariates

We characterized beneficiary-level covariates, including age at diagnosis, race and ethnicity (Hispanic, non-Hispanic Black, non-Hispanic White, and other or unknown),^27,28^ stage at diagnosis, cancer type, census tract–level socioeconomic status (SES; Yost Index quintile),^29,30^ and urbanicity.^31^ To describe the representativeness of the sample and examine disparities in plan switching, we report race and ethnicity based on the Research Triangle Institute race code on Medicare enrollment records, which supplement information sourced from Social Security Administration enrollment with an imputation algorithm. We included beneficiaries identified as Asian or Pacific Islander and American Indian or Alaska Native in the other or unknown category given their small sample sizes. Based on the beneficiary’s last known MA plan, we linked publicly available plan-level characteristics, including plan type (eg, health maintenance organization [HMO], preferred provider organization [PPO]), out-of-network coverage, monthly premiums (in quartiles but separating out plans with zero premiums), and star ratings.^32^

### Statistical Analysis

We conducted a difference-in-differences analysis to examine differential changes in the probability of plan switching between beneficiaries diagnosed in states with and without guaranteed issue protections, before and after the year of cancer diagnosis (T_0_). The model included beneficiary-level covariates, county fixed effects (functionally absorbing any fixed state-level confounders), and calendar-year fixed effects (adjusting for secular trends in plan switching). We clustered standard errors at the state level because the exposure to guaranteed issue protections varied by state. To elucidate dynamic changes in plan switching and assess prediagnosis trends, we also estimated an event study model showing differential change in the outcome for each year relative to the reference year (T_−1_). In addition, we explored the heterogeneity in the main difference-in-differences estimate by stratifying the model by each level of covariate. Data were analyzed using SAS Studio (SAS Institute) and Stata, version 14 (StataCorp). We used α = .05 to assess statistical significance.

### Supplementary Analyses

We include several sets of supplementary analyses. First, we conducted a falsification test, using beneficiaries continuously enrolled in full Medicaid (hereafter, duals) as a placebo group. As Medicaid largely covers Medicare cost sharing, duals are presumably unaffected by state Medigap policies in making the decision to switch to TM.^14^ The test may illuminate other time-varying confounders (eg, MA network restrictions) that may impact older adults’ decisions to switch to TM after a diagnosis. To further help rule out other changes in local characteristics associated with plan switching, we (1) tested the sensitivity of the model to adjusting for county-specific linear trends, (2) conducted a triple difference-in-differences model that included a random sample of Medicare beneficiaries without cancer as an additional within-county control, and (3) limited the sample to SEER states in the Northeast (Connecticut, Massachusetts, New York, and New Jersey) to minimize regional differences.

Second, the analysis may be confounded by the compositional changes in diagnosed cases between states with and without guaranteed issue protections. For example, differential mortality may be an important confounder, given that early deaths are common among patients with cancer. Thus, we investigated mortality rates by states and the robustness of the model to including patients who died prior to T_1_. Furthermore, endogenous mobility (ie, beneficiaries moving to guaranteed issue states), in anticipation of or following diagnosis, may cross contaminate the sample. To rule this out, we used an alternative exposure variable based on the initial county of residence at T_−4_, rather than the county at diagnosis, which is less likely to be confounded. Lastly, as a general check for other compositional changes, we examined trends in the measured covariates. Third, we accounted for the small cluster of guaranteed issue states (N = 3), as well as outlier states, by performing a randomization inference (testing the significance of the main estimate against a distribution of pseudorandom estimates^33^) and a leave-one-out analysis that may reveal influential states. Data were analyzed from October 2024 to April 2025.

## Results

### Sample Characteristics

The sample included 180 075 MA beneficiaries 69 years and older who were newly diagnosed with cancer from 2014 to 2019 (Table 1). Among these beneficiaries, 44.5% were diagnosed between the ages of 69 and 75 years, 51.5% were male, 8.0% were Hispanic, 7.4% were non-Hispanic Black, 78.5% were non-Hispanic White, and 6.1% were of another or unknown race and ethnicity. There were several differences in the measured characteristics between beneficiaries diagnosed in states with and without guaranteed issue rights (standardized mean difference, >0.15). Beneficiaries in guaranteed issue states were more likely to be non-Hispanic White, reside in higher SES quintiles, and be enrolled in MA plans with broader networks (ie, non-HMO) and higher premiums but lower star ratings (Table 1).

**Table 1.** Characteristics of Medicare Advantage (MA) Beneficiaries Newly Diagnosed With Cancer in States With and Without Guaranteed Issue Rights^a^

Characteristic	Participants, No. (%)			
	Overall (N = 180 057)	Diagnosed in guaranteed issue states (n = 41 786)	Diagnosed in all other states (n = 138 271)	Standardized mean difference
Sex^b^				
Female	87 309 (48.5)	20 328 (48.6)	66 981 (48.4)	0.004
Male	92 748 (51.5)	21 458 (51.4)	71 290 (51.6)	−0.004
Race and ethnicity^c^^,^^d^				
Hispanic	14 354 (8.0)	1607 (3.8)	12 747 (9.2)	0.012
Non-Hispanic White	141 309 (78.5)	35 455 (84.8)	105 854 (76.6)	0.199
Non-Hispanic Black	13 411 (7.4)	3202 (7.7)	10 209 (7.4)	−0.197
Other or unknown	10 983 (6.1)	1522 (3.6)	9461 (6.8)	−0.132
Age at diagnosis, y^b^				
69-75	80 086 (44.5)	18 208 (43.6)	61 878 (44.8)	−0.023
76-85	79 509 (44.2)	18 915 (45.3)	60 594 (43.8)	0.028
86-100	20 462 (11.4)	4663 (11.2)	15 799 (11.4)	−0.008
Stage at diagnosis^b^				
In situ/localized	107 006 (59.4)	24 569 (58.8)	82 437 (59.6)	−0.015
Regional	30 172 (16.8)	6964 (16.7)	23 208 (16.8)	−0.004
Distant	33 578 (18.6)	7844 (18.8)	25 734 (18.6)	0.003
Unstaged	9301 (5.2)	2409 (5.8)	6892 (5.0)	0.035
Cancer sites^b^				
Breast	36 221 (20.1)	8141 (19.5)	28 080 (20.3)	−0.021
Colorectal	17 494 (9.7)	3955 (9.5)	13 539 (9.8)	−0.011
Lung	23 199 (12.9)	5903 (14.1)	17 296 (12.5)	0.048
Prostate	32 536 (18.1)	7877 (18.9)	24 659 (17.8)	0.027
Other	70 607 (39.2)	15 910 (38.1)	54 697 (39.6)	−0.031
Yost Index quintile^b^^,^^e^				
1 (Lowest SES)	18 635 (10.3)	2555 (6.1)	16 080 (11.6)	−0.181
2	25 247 (14.0)	4924 (11.8)	20 323 (14.7)	−0.084
3	34 414 (19.1)	8373 (20.0)	26 041 (18.8)	0.029
4	44 527 (24.7)	11 739 (28.1)	32 788 (23.7)	0.102
5 (Highest SES)	52 987 (29.4)	12 301 (29.4)	40 686 (29.4)	0.001
Urbanicity^b^^,^^f^				
Metro	166 045 (92.2)	38 739 (92.7)	127 306 (92.1)	0.025
Nonmetro	14 012 (7.8)	3047 (7.3)	10 965 (7.9)	−0.025
Year of diagnosis^b^				
2014	25 224 (14.0)	6411 (15.3)	18 813 (13.6)	0.050
2015	27 163 (15.1)	6466 (15.5)	20 697 (15.0)	0.015
2016	28 521 (15.8)	6575 (15.7)	21 946 (15.9)	−0.003
2017	31 657 (17.6)	7094 (17.0)	24 563 (17.8)	−0.022
2018	33 018 (18.3)	7564 (18.1)	25 454 (18.4)	−0.008
2019	34 474 (19.1)	7676 (18.4)	26 798 (19.4)	−0.026
MA plan type^d^				
HMO	116 144 (64.5)	22 749 (54.4)	93 395 (67.5)	−0.271
POS	9324 (5.2)	5965 (14.3)	3359 (2.4)	0.528
PFFS	2524 (1.4)	740 (1.8)	1784 (1.3)	0.040
Local PPO	41 343 (23.0)	9348 (22.4)	31 995 (23.1)	−0.019
Regional PPO	8878 (4.9)	2880 (6.9)	5998 (4.3)	0.116
Other^g^	1844 (1.0)	104 (0.2)	1740 (1.3)	−0.100
OON coverage^d^				
Yes	52 049 (28.9)	12 826 (30.7)	39 223 (28.4)	0.050
No	128 008 (71.1)	28 960 (69.3)	99 048 (71.6)	−0.050
Premium amount^d^				
Zero premium	82 906 (46.0)	16 378 (39.2)	66 528 (48.1)	−0.177
Quartile 1 (≤$20.80)	17 926 (10.0)	5290 (12.7)	12 636 (9.1)	0.117
Quartile 2 ($20.81-$43.80)	17 878 (9.9)	5495 (13.2)	12 383 (9.0)	0.137
Quartile 3 ($43.81-$74.10)	17 571 (9.8)	3469 (8.3)	14 102 (10.2)	−0.064
Quartile 4 (>$74.10)	18 100 (10.1)	6414 (15.3)	11 686 (8.5)	0.226
Star rating^d^				
2-2.5	1027 (0.6)	222 (0.5)	805 (0.6)	−0.007
3-3.5	47 418 (26.3)	14 393 (34.4)	33 025 (23.9)	0.240
4-4.5	95 705 (53.2)	24 243 (58.0)	71 462 (51.7)	0.123
5	32 999 (18.3)	2479 (5.9)	30 520 (22.1)	−0.416
Missing	2908 (1.6)	449 (1.1)	2459 (1.8)	−0.048

Abbreviations: HMO, health maintenance organization; OON, out of network; PFFS, private fee for service; POS, point of service; PPO, preferred provider organization; SES, socioeconomic status.

^a^ Characteristics of MA beneficiaries newly diagnosed with cancer in states with guaranteed issue rights for Medigap in the Surveillance, Epidemiology, and End Results Program (Connecticut, Massachusetts, and New York) and other remaining states (California, Georgia, Hawaii, Idaho, Iowa, Kentucky, Louisiana, Michigan, New Jersey, New Mexico, Texas, Utah, and Washington) from 2014 to 2019 meeting the inclusion criteria.

^b^ Variables extracted from the Surveillance, Epidemiology, and End Results Program files.

^c^ Variables extracted from Medicare Master Summary Beneficiary File.

^d^ Based on Research Triangle Institute race code. Beneficiaries identified as Asian or Pacific Islander and American Indian or Alaska Native were included in the other or unknown category given their small sample sizes.

^e^ Surveillance, Epidemiology, and End Results Program summary stage at diagnosis.

^d^ Characteristics of the last known MA plan for each beneficiary that were obtained from publicly available plan benefits package and star ratings data.

^e^ Yost Index is a census tract–level measure of SES, with a lower quintile representing tracts with the lowest SES.

^f^ Urbanicity was categorized based on the county-level Rural-Urban Commuting Area Codes.

^g^ Includes §1876 and §1833 cost plans.

### Changes in Plan Switching

**Figure 1.**
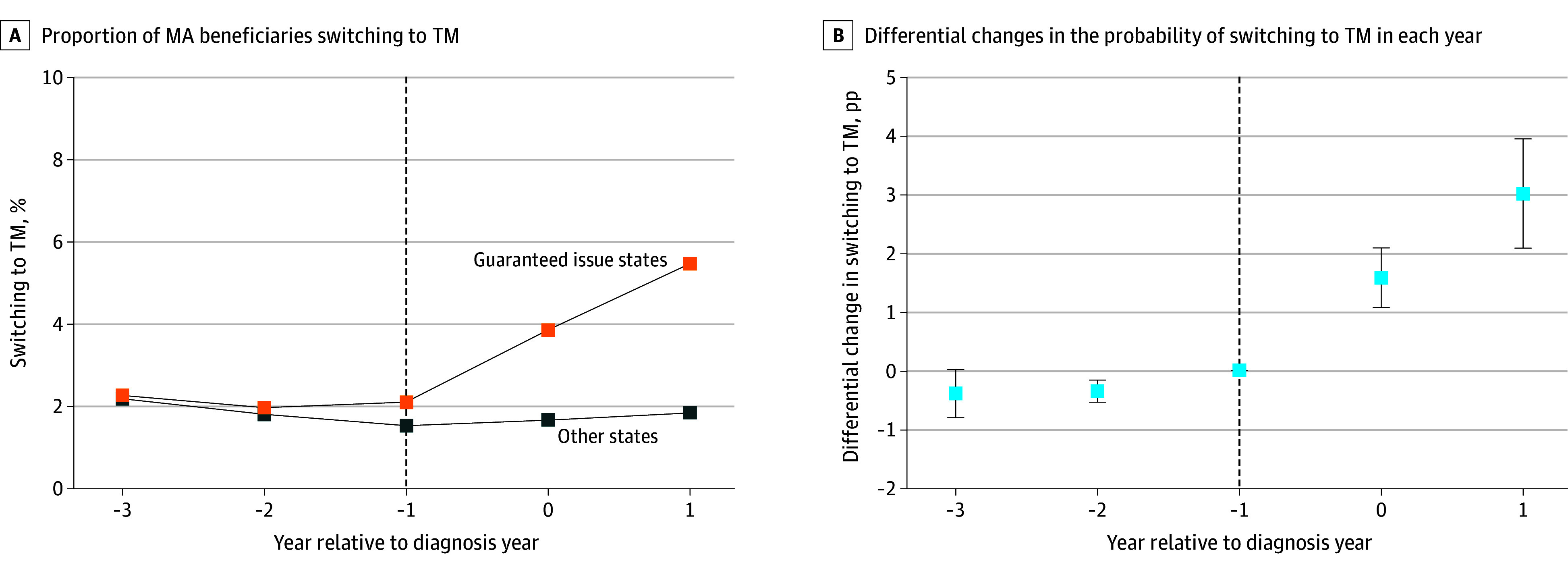
Trends and Changes in Medicare Advantage (MA) Disenrollment Between Beneficiaries Newly Diagnosed With Cancer in States With and Without Guaranteed Issue Rights A, The unadjusted proportion of MA beneficiaries switching to traditional Medicare (TM) in each normalized year relative to the diagnosis year for those diagnosed in states with and without guaranteed issue rights. Plan switching was examined only once, and beneficiary-year observations after the switch are censored from the denominator. The dashed vertical line corresponds to the reference year (ie, the year prior to the diagnosis year). B, Adjusted event study coefficients (in percentage points [pp]) are plotted showing differential changes in the probability of switching to TM in each year, relative to the reference year (dashed vertical line). The model adjusted for county and year fixed effects and beneficiary-level controls, with clustered standard errors at the state level. Error bars indicate 95% CIs.

Among beneficiaries diagnosed in guaranteed issue states, the rate of switching to TM was 2.1% before diagnosis and 4.7% after cancer diagnosis, whereas the rate of switching remained unchanged in other states (1.8% to 1.7%) (Figure 1A and Table 2). This corresponded to a difference-in-differences of 2.5 percentage points (pp; 95% CI, 1.9-3.2 pp; *P* < .001), representing more than a 120% relative increase from the baseline switching probability (2.1%). The estimate was robust to the inclusion of beneficiary-level covariates and county-specific linear trends. The event study estimates suggest no clear trends in switching in the prediagnosis period and show a larger differential switching in the year following diagnosis (3.0 pp; 95% CI, 2.1-3.9 pp; *P* < .001), rather than the year of diagnosis (1.6 pp; 95% CI, 1.1-2.1 pp; *P* < .001) (Figure 1B).

**Table 2.** Differential Changes in the Probability of Medicare Advantage Disenrollment Among Beneficiaries Diagnosed in States With and Without Guaranteed Issue Rights, Before and After Cancer Diagnosis

Variable	Model 1	Model 2	Model 3
Difference-in-differences (95% CI), pp^a^	2.56 (1.85-3.27)	2.53 (1.86-3.21)	2.09 (1.38-2.80)
*P* value	<.001	<.001	<.001
Prediagnosis switching, %^b^	2.1	2.1	2.1
% Change^c^	121	120	99
County fixed effects	Yes	Yes	Yes
Calendar-year fixed effects	Yes	Yes	Yes
Beneficiary-level controls^d^	No	Yes	Yes
County-specific linear trend	No	No	Yes

Abbreviation: pp, percentage point.

^a^ The difference-in-differences estimates show changes in the probability of switching between Medicare Advantage beneficiaries 69 years and older diagnosed in states with guaranteed issue rights, relative to contemporaneous changes among those diagnosed in other states, before and after cancer diagnosis. Standard errors were clustered at the state level. Model 2 is the main specification reported in text.

^b^ Prediagnosis rate of switching to traditional Medicare among beneficiaries diagnosed in states with guaranteed issue rights.

^c^ The relative change was calculated as the difference-in-differences estimates divided by the prediagnosis switching rate.

^d^ Beneficiary-level controls included all covariates from Table 1, except the urbanicity variable, which was collinear with county fixed effects.

**Figure 2.**
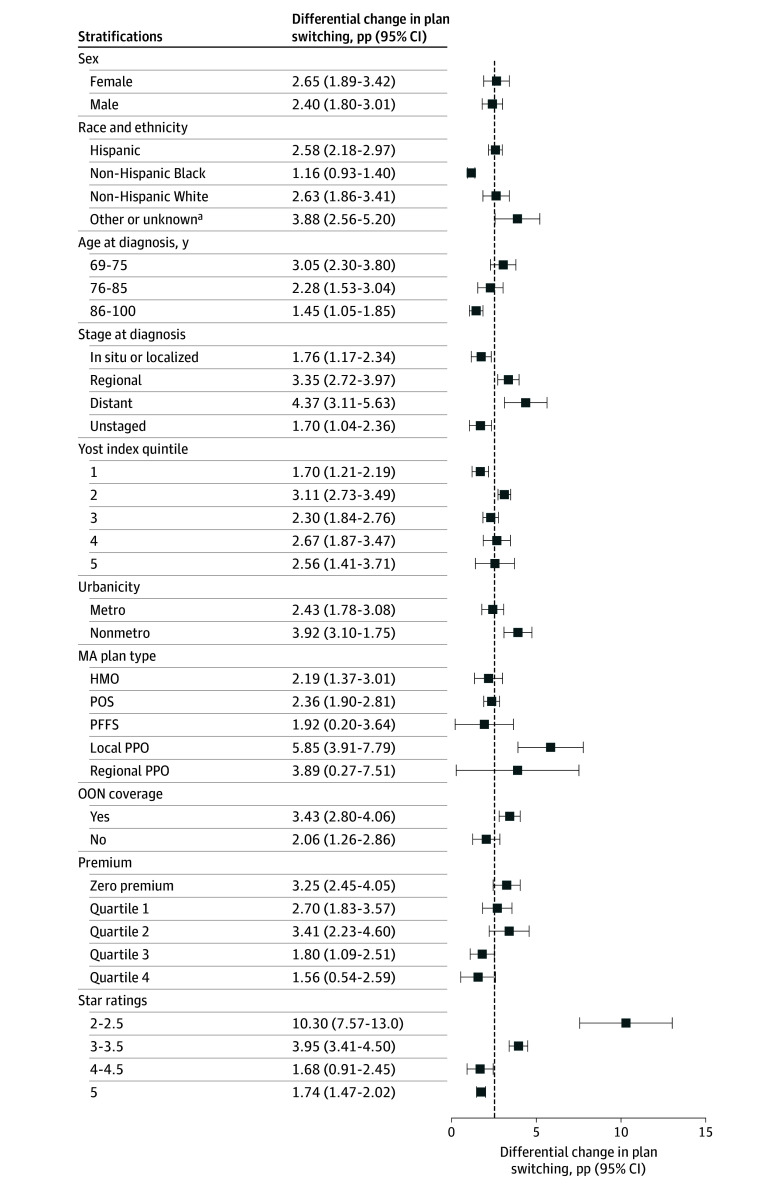
Differential Changes in Medicare Advantage Disenrollment Among Beneficiaries by Stratifications Each row plots the adjusted difference-in-differences coefficient (in percentage points [pp]), estimated among the listed stratification. The dashed vertical line corresponds to the magnitude of the difference-in-differences coefficient from the main model (β, 2.53). All estimates were statistically significant at the α = .05 level. The model adjusted for county and year fixed effects and beneficiary-level controls, with clustered standard errors at the state level. The full coefficients are available in eTable 1 in Supplement 1. HMO indicates health maintenance organization; MA, Medicare Advantage; OON, out of network; PFFS, private fee for service; POS, point of service; PPO, preferred provider organization. ^a^Owing to small sample sizes, the other or unknown category includes beneficiaries identified as Asian or Pacific Islander and American Indian or Alaska Native.

Heterogeneity was noted in differential switching by key covariates (Figure 2). In terms of demographics, there was a greater differential switch among beneficiaries who were younger, non-Hispanic White, and residing in nonmetro and higher SES quintile areas. Moreover, larger changes were observed among those diagnosed with more advanced stage cancer or cancer with poor prognosis (eg, brain or pancreatic cancers; eTable 1 in Supplement 1). Finally, those enrolled in plans with more comprehensive coverage (ie, PPO plans or plans with out-of-network coverage) and lower star-rating plans (2-3.5 stars) experienced greater differential disenrollment in guaranteed issue states (Figure 2).

### Supplementary Analyses

The falsification test did not detect statistically significant differential changes in the probability of switching among duals (eFigure 2 in Supplement 1). The estimate was robust to including noncancer controls and limiting the sample to the Northeast (eFigure 3 and eTable 2 in Supplement 1). Any differential changes in the measured characteristics of the sample were ruled out, including mortality (eTables 3 and 4 in Supplement 1), endogenous mobility (eTable 5 in Supplement 1), and other covariates (eTable 6 in Supplement 1). The randomization inference test yielded a similar level of statistical significance for the main difference-in-differences estimate (eFigure 4 in Supplement 1), and the leave-one-out analysis revealed no influential states (eFigure 5 in Supplement 1).

## Discussion

In a large, nationally representative sample of MA beneficiaries newly diagnosed with cancer, we found a sharp increase in rates of switching to TM following diagnosis, almost twice the switching rate among the general population (2%-3% in recent periods^34,35^). However, this increase was observed predominantly in states providing Medigap guaranteed issue protections, suggesting that MA beneficiaries in other states may have had limited options for TM coverage due to difficulties in obtaining Medigap coverage.

Prior to this work, to our knowledge, only 2 studies have evaluated the associations of state Medigap laws with plan switching among MA beneficiaries.^15,36^ Liu et al^15^ examined switching among patients newly initiating top highest-spending Part B drugs (including those for cancer), finding a 3.7-pp higher probability of MA disenrollment in guaranteed issue states. Among initially enrolled MA beneficiaries who then switched into TM, Meyers et al^36^ documented a 16.9-pp higher reenrollment rate in MA in states without Medigap consumer protections. In contrast, the present study focused on the role of a new cancer diagnosis (ie, a substantial health shock generating an impetus for plan switching) and longitudinally assessed the trends of plan switching, while incorporating important associated factors of plan switching such as MA plan, demographic, and fixed area-level characteristics.

MA beneficiaries with cancer are particularly at risk for high switching costs for 2 reasons. First, compared to other chronic conditions, cancer diagnosis is often unanticipated^37,38^ and complicates the ability to make an optimal plan selection prospectively. Because Medicare beneficiaries are only allowed to switch between MA and TM during the open enrollment period,^39^ most beneficiaries diagnosed outside of this period may be locked in to their coverage. This may explain the finding that even in guaranteed issue states, we observed a greater rate of switching in the year following the diagnosis year, rather than the diagnosis year. Second, cancer diagnosis generates an acute need for complex and specialized care,^40,41^ but meeting such needs may be challenging in MA due to coverage denials, prior authorizations, and narrow networks.^6,18^ This may explain more pronounced disenrollment among beneficiaries diagnosed with advanced-stage or rare cancers (eg, brain cancer), which necessitate more complex oncologic care. Furthermore, we note a particularly high share of disenrollment among beneficiaries in plans with lower star ratings, which may suggest poor care quality and/or higher discontinuation of plans by insurers. These findings contribute to the growing evidence of dissatisfaction and disenrollment from MA among high-cost and high-needs beneficiaries, such as beneficiaries with Alzheimer or end-stage kidney disease.^42,43,44,45^

Interestingly, beneficiaries enrolled in plans with broader networks (such as PPOs) that may provide wider access to clinicians on average were more likely to disenroll in guaranteed issue states. One interpretation of this finding may suggest adverse selection in plan enrollment.^46,47^ High-cost and high-needs beneficiaries, who may be more likely to shop around for plans, may have been disproportionately enrolled in plans with broader networks prior to diagnosis, but they are also generally more likely to disenroll from MA.^42,43,44,45^ Furthermore, beneficiaries who could afford more generous plans may have higher SES,^48^ making them less price sensitive to the cost of switching to TM even if they are unable to purchase supplemental coverage. On the other hand, differences in care quality may explain findings of this study. For example, prior studies have shown that HMOs often performed better on quality measures than PPOs,^49,50^ suggesting that HMOs may have delivered better care by leveraging care coordination through narrow networks.^49,50,51^ This may mean that conditional on being in MA, beneficiaries may have been more satisfied with the quality of these plans. However, the fact that beneficiaries across all plan types were more likely to disenroll from MA in guaranteed issue states raises an overarching question regarding the appropriateness of managed care for optimal cancer care delivery, especially in areas where plans may have limited abilities to form high-quality networks (eg, rural areas^52,53^). With evidence shedding light on barriers to cancer care in MA, such as limited access to accredited cancer facilities^51,54^ and denials of care,^18,55,56^ robust research investigating the quality of cancer care in MA and evolving changes in plan quality over time is warranted to better understand the drivers of plan switching in this population.

It is important to note that high premiums in Medigap impose barriers to supplemental coverage, even in states providing guarantee issue protections. In 2023, the average annual premium for Medigap was $2604,^57^ putting Medigap out of reach for many older adults with limited incomes.^10^ We observed sociodemographic disparities in plan switching that may reflect cost-related barriers to Medigap, with lower rates of switching among beneficiaries who are impacted by income and wealth inequality (eg, those who are non-Hispanic Black, older, and living in lower socioeconomic areas). For these beneficiaries, the cost of Medigap premiums may weigh heavily against the decision to switch to TM, even when TM coverage may be optimal for care.^57,58^

One solution to addressing the inaccessibility of Medigap is to expand guaranteed issue protections in all states. However, there are important trade-offs to be considered. Expanded guarantee issue rights may worsen adverse selection by further shifting high-cost beneficiaries into the TM and Medigap pool, which may raise premiums and price out marginal beneficiaries facing income constraints.^59^ Moreover, increased reliance on TM to provide the bulk of specialty care may further accelerate the segmentation of Medicare into 2 pools,^60^ reducing the incentive for MA plans to improve care quality and exacerbating existing problems with the MA program, such as plan overpayments.^1,60^ Additionally, attaining Medigap coverage may engender increased utilization and health care spending by lowering the out-of-pocket cost burden (ie, moral hazard).^61,62,63^ These trade-offs demonstrate the need to approach Medigap reforms through a more holistic lens. For instance, a comprehensive reform to Medicare’s benefit design may be needed to address the shortfalls of TM and MA coverage in parallel (eg, instituting an out-of-pocket maximum in TM but subsidize it by reducing MA payments^59^), which may obviate the need to rely on Medigap as the backstop to catastrophic care costs in TM.

### Limitations

We note several limitations. We are unable to isolate the causal effects of state guaranteed issue protections, as they remained static over time. In light of other unmeasured confounders, we cautiously interpret these estimates as associations of state guaranteed issue protections with plan switching. Furthermore, we could not ascertain actual Medigap enrollment in administrative Medicare data, which do not include information on supplemental coverage, though we hypothesize that many beneficiaries who disenrolled from MA likely have obtained Medigap to cover cost sharing in TM.^64^ Lastly, we are unable to ascertain disenrollment for other reasons, such as contract terminations.

## Conclusions

In this cohort study, state Medigap guaranteed issue protections were associated with higher rates of switching to TM among MA beneficiaries newly diagnosed with cancer. Reforms to address the catastrophic out-of-pocket costs and limited access to Medigap in TM, while improving the delivery of specialty care in MA, are needed to ensure that Medicare beneficiaries with serious health conditions can affordably access high-quality care.

## Notes

Supplement 1.
eMethods
**eFigure 1.** Sample Derivation Flowchart **eTable 1.** Full stratified analysis results **eFigure 2.** Falsification test using MA beneficiaries who were dually enrolled in Medicaid **eFigure 3.** Difference in difference-in-differences between beneficiaries diagnosed with and without cancer **eTable 2.** Sensitivity to limiting the sample to the SEER states in the Northeast **eTable 3.** Mortality rates by states with and without guaranteed issue rights **eTable 4.** Sensitivity of main difference-in-differences estimate to including beneficiary-year observations among those who died in the year of cancer diagnosis **eTable 5.** Sensitivity of main difference-in-differences estimate to endogenous mobility **eTable 6.** Differential changes in other measured covariates **eFigure 4.** Distribution of pseudo-random estimates from randomization inference test **eFigure 5.** Results of “leave-one-out” analysis
eReferences

Supplement 2.
Data Sharing Statement
