# Supplementary material for: Medigap Protection and Plan Switching Among Medicare Advantage Enrollees With Cancer
Source: JAMA Health Forum. 2025 Jun 7;6(6):e252018. doi: 10.1001/jamahealthforum.2025.2018 (PMC12145528; doi:10.1001/jamahealthforum.2025.2018)
Supplement: Supplement 1. — eMethods eFigure 1. Sample Derivation Flowchart eTable 1. Full stratified analysis results eFigure 2. Falsification test using MA beneficiaries who were dually enrolled in Medicaid eFigure 3. Difference in difference-in-differences between beneficiaries diagnosed with and without cancer eTable 2. Sensitivity to limiting the sample to the SEER states in the Northeast eTable 3. Mortality rates by states with and without guaranteed issue rights eTable 4. Sensitivity of main difference-in-differences estimate to including beneficiary-year observations among those who died in the year of cancer diagnosis eTable 5. Sensitivity of main difference-in-differences estimate to endogenous mobility eTable 6. Differential changes in other measured covariates eFigure 4. Distribution of pseudo-random estimates from randomization inference test eFigure 5. Results of “leave-one-out” analysis eReferences [file jamahealthforum-e252018-s001.pdf]

Supplemental Online Content

Kwon Y, Jazowski SA, Hu X, et al. Medigap protection and plan switching among Medicare Advantage enrollees with cancer. *JAMA Health Forum*. 2025;6(6):e252018. doi:10.1001/jamahealthforum.2025.2018

eMethods
eFigure 1. Sample Derivation Flowchart
eTable 1. Full stratified analysis results
eFigure 2. Falsification test using MA beneficiaries who were dually enrolled in Medicaid
eFigure 3. Difference in difference-in-differences between beneficiaries diagnosed with and without cancer
eTable 2. Sensitivity to limiting the sample to the SEER states in the Northeast
eTable 3. Mortality rates by states with and without guaranteed issue rights
eTable 4. Sensitivity of main difference-in-differences estimate to including beneficiary-year observations among those who died in the year of cancer diagnosis
eTable 5. Sensitivity of main difference-in-differences estimate to endogenous mobility
eTable 6. Differential changes in other measured covariates
eFigure 4. Distribution of pseudo-random estimates from randomization inference test
eFigure 5. Results of "leave-one-out" analylsis
eReferences

This supplemental material has been provided by the authors to give readers additional information about their work.

eMethods.

Description of SEER-Medicare Data

The SEER-linked Medicare data captures nearly all incident cancer cases among participating state cancer registries and provides their Medicare enrollment information, which are linked to eligible cancer cases based on a deterministic algorithm.[1] Over 96% of cancer cases among individuals aged 65 or older in SEER are linked to their Medicare data. Our data encompassed cases for 14 common cancer sites (breast, brain and other nervous systems colorectal, lung, prostate, leukemia, liver, lung, lymphoma, melanoma, multiple myeloma, ovary, pancreas, and prostate) that represent nearly 81% of all incident cancer cases in the United States.[2] The following SEER registries were included in our data: California, Connecticut, Georgia, Hawaii, Idaho, Iowa, Kentucky, Louisiana, Massachusetts, Detroit, New Jersey, New Mexico, New York, Texas, Utah, and Seattle. Our SEER data captured new cancer cases who were diagnosed from 2010 to 2019. We utilized the linked Medicare enrollment files for each eligible cancer case, which provide all available Medicare enrollment records for the linked cases, before and after cancer diagnosis. Thus, the data allows us to longitudinally examine changes in enrollment in Medicare Advantage (MA) vs. Traditional Medicare (TM) for each Medicare beneficiary newly diagnosed with cancer.

Sample Derivation Process

eFigure 1 details how we derived the study sample. We first identified 1,708,440 primary cancer cases (i.e., the first lifetime cancer diagnosis), using the sequence number variable (Sequence number = 00 or 01). A small portion of the cases (4.2%; N=71,030) had duplicate records across cancer files. For these cases, we randomly retained one case record to create a sample of unique cancer cases. We then applied several demographic and enrollment-related

© 2025 Kwon Y et al. *JAMA Health Forum*.

exclusion criteria. We excluded those who were diagnosed at death or autopsy (using the reporting source variable in SEER; N=39,334 or 2.4%), those who were eligible due to disability or end-stage renal disease (N=271,666, 16.6%) or dually enrolled with Medicaid at any point during the study window (N=211,760, 12.9%), those diagnosed with cancer before age 69 (N=379,123, 23.2%), those without continuous enrollment in Medicare Part A and B (N=71,752, 4.4%), and those who died in the year of diagnosis (N=108,220, 6.6%). After exclusions, there were 555,555 aged Medicare beneficiaries newly diagnosed with cancer, 32.4% of whom were initially enrolled in MA (i.e., in the 4th year before the diagnosis year). To categorize beneficiaries who were diagnosed in guaranteed issue states vs. other states, we used the state at diagnosis in the SEER data. The final sample of MA beneficiaries included 41,786 (199,526 beneficiary-years) and 138,271 (665,572 beneficiary-years) diagnosed in the guaranteed issue states and other states in the SEER registries, respectively.

Construction of enrollment panel data

We followed a prior approach that was used to examine the effects of cancer diagnosis on plan switching.[3] For each Medicare beneficiary in the sample, we created a longitudinal panel of enrollment data for 4 years before diagnosis ($Year = T_{-4}$) and 1 year after diagnosis (T_1). We identified beneficiaries who were initially enrolled in MA for 12 months in T_{-4} and then tracked their plan choice from T_{-3} to T_1. For years T_{-3} to T_1, we constructed a binary variable that was 1 for the beneficiary-year when a beneficiary disenrolled from MA. Plan selection for a given year is identified in December for most cases, but for those who were not alive through December in T_1, we used the enrollment information in the month of death. For simplicity, we observed plan switching only once, following each beneficiary from T_{-3} (i.e., the first year in which we can observe any switching) until the point of disenrollment or through T_1.

© 2025 Kwon Y et al. *JAMA Health Forum*.

eFigure 1. Sample Derivation Flowchart

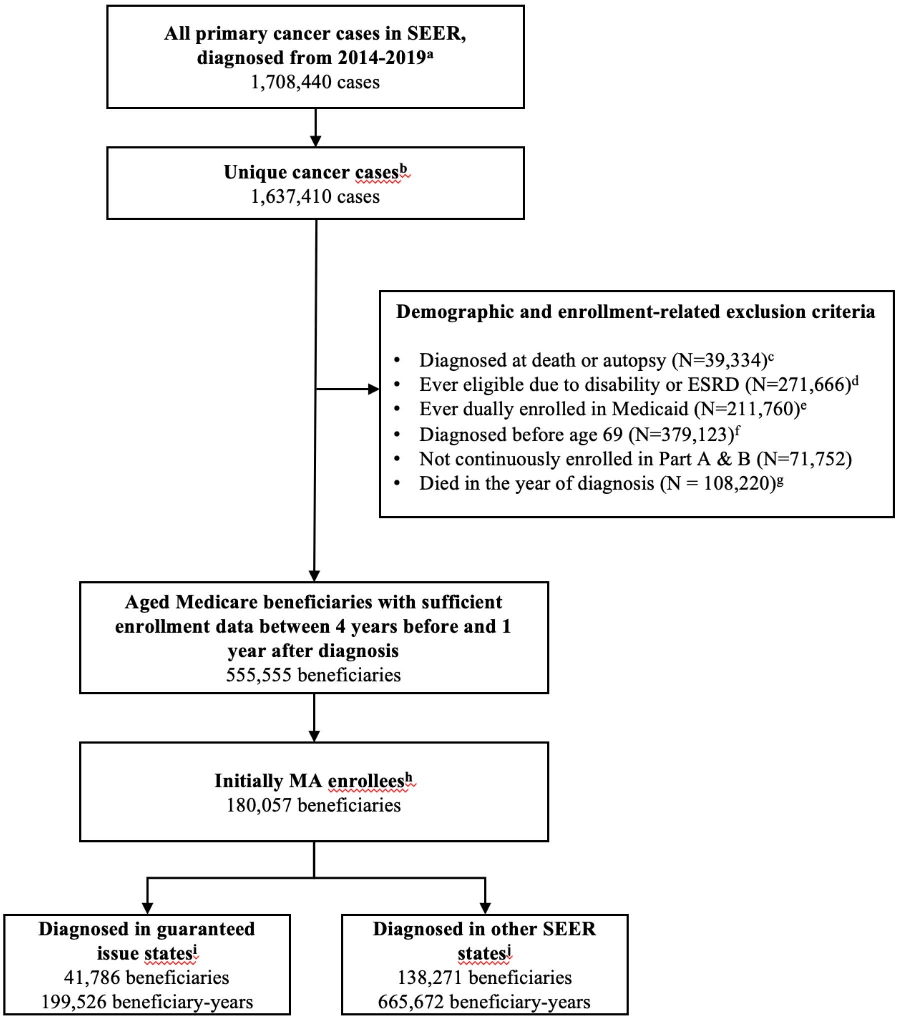

Abbreviations: SEER, Surveillance, Epidemiology, and End Results Program; ESRD, end-stage renal disease

[a] All primary cancer cases (i.e., the first cancer diagnoses) for the following cancer sites: bladder, brain and nervous system, breast, colorectal, kidney, leukemia, liver, lung, lymphoma, melanoma, multiple myeloma, ovary, pancreas, and prostate

[b] We excluded a small portion of cancer cases that appeared in multiple cancer specific files (N=142,727). For those appearing across files, we randomly picked a diagnosis to include.

[c] We identified cancers diagnosed at death and autopsy using the type of reporting source variable.

[d] We excluded those who were eligible for disability or ESRD at any point during the enrollment window

[e] We excluded those with any partial (i.e., Medicare Savings Programs) dual coverage or full Medicaid coverage during the enrollment window.

[f] We required at least 4 years of enrollment prior to cancer diagnosis, which excluded those diagnosed before age 69.

[g] To observe plan switching in the year following diagnosis, we required that beneficiaries be alive at least through the January in the year after diagnosis.

[h] We determined initial MA enrollees based on MA enrollment for 12 months in the fourth year prior to the diagnosis year.

[k] We used the state at diagnosis variable in the SEER data to identify those diagnosed in states with guaranteed issue rights represented in SEER (Connecticut, Massachusetts, and New York); all other states in SEER included (California, Georgia, Hawaii, Idaho, Iowa, Kentucky, Louisiana, Michigan, New Jersey, New Mexico, Texas, Utah, and Washington).

© 2025 Kwon Y et al. *JAMA Health Forum*.

Identification Strategy: difference-in-differences estimation

To evaluate the associations of state Medigap guaranteed issue rights with MA disenrollment, we implemented a difference-in-differences (DD) design comparing a person-level probability of disenrollment between those diagnosed in states with vs. without guaranteed issue rights following diagnosis. We estimated the following linear probability DD model:

$$y_{ict} = \alpha + \beta_1 GuaranteedIssue_{ic} * Post_{it} + \beta X_i + \delta_c + \gamma_t + \varepsilon_{ict}$$

where y_{it} is the switching outcome for a beneficiary i, diagnosed in county c, in year t (normalized to diagnosis year). $GuaranteedIssue_{ic}$ is 1 for beneficiaries diagnosed in guaranteed issue states, and $Post_{it}$ is 1 for beneficiary-year observations in T_0 and T_1 (i.e., the diagnosis year and the following year). Our model adjusted for county fixed effects (δ_c) to account for fixed county-level differences in the outcome (e.g., regulatory and MA plan landscape) and calendar year fixed effects (γ_t) to adjust for secular trends in switching. We controlled for a vector of person-level confounders (X_i) to improve the precision of our estimates. We cluster standard errors at the state-level at which there is variation in guaranteed issue rights.

To elucidate dynamic changes in switching and to test for the parallel trends assumption of DD, we also estimated an event study form, as shown below:

$$y_{ict} = \alpha + \sum_{j=-3, j\neq-1}^{1} \beta_j I(t = T_j) * GuaranteedIssue_{ic} + \beta X_i + \delta_c + \gamma_t + \varepsilon_{ict}$$

In this model, we replaced the $Post_{it}$ term in the prior equation with a series of normalized year dummies $I(t = T_j)$ for normalized years $j \in \{-3, -2, 0, 1\}$, taking the year prior to diagnosis ($j = -1$) as the reference year. We test that β_{-3} and β_{-2} are statistically indistinguishable from zero to demonstrate there are no significant pre-trends in the outcome variable.

© 2025 Kwon Y et al. *JAMA Health Forum*.

Sensitivity Analyses

We executed multiple sets of sensitivity analyses to confirm the robustness of our findings. We conducted a falsification test using beneficiaries who were continuously enrolled in Medicaid (either full Medicaid or full Medicaid coverage of cost-sharing through Medicare Savings Program[4]) as a placebo group. For duals, Medigap carriers are not able to offer coverage,[5] as Medicaid fully covers their Medicare cost-sharing. Therefore, access to Medigap is a less of a factor in making the decision to switch to TM, and we would expect a null finding in this group. This test can illuminate other time-varying confounders (such as MA network restrictions or provider nudges to disenroll from MA) that may impact our DD estimates.

To rule out other local changes that may be correlated with plan switching, we conducted the following analyses. First, we re-estimated our main model but included county x. continuous year fixed effects, to better capture county-specific linear trends which could impact switching to TM. Second, we conducted a triple difference-in-differences model, using a sample of Medicare beneficiaries without a cancer diagnosis in each county as an additional control group. This cohort is provided as part of the SEER-Medicare linkage, which includes a random 5% sample of persons with no known history of cancer diagnosis.[6] For this group, we assigned a "pseudo-diagnosis date" by arbitrarily selecting a date during which they were enrolled in Medicare and constructed the study cohort similarly as our main cohort. Third, we replicated our main model, but just among SEER states in the Northeast, three of which are guaranteed issue states (Connecticut, Massachusetts, and New York), with New Jersey serving as the main control group, to minimize regional variation in switching and generate more comparable units for the analysis.

© 2025 Kwon Y et al. *JAMA Health Forum*.

Other key source of bias is the compositional changes among diagnosed cases between states with and without guaranteed issue protections that have time-varying effects. In the context of our setting, we are especially concerned about two factors. First, there may be differential attrition due to early mortality, which may change the characteristics of the beneficiary-year observations for the year following diagnosis year (T_1). Thus, we investigated rates of 1-year mortality and conducted a sensitivity analysis where we include cases who died in the year of diagnosis (i.e., an unbalanced panel). Second, endogenous mobility (i.e., beneficiaries moving to a guaranteed issue state) in anticipation of or following cancer diagnosis would cross-contaminate the sample. To rule this out, we assessed mobility as an outcome (changes in county of residence in Medicare enrollment files) and replicated our model but use the initial county of residence to assign the exposure. Finally, we examined trends in measured covariates during the enrollment window (T_{-3} to T_1), which may reveal other compositional changes in the sample.

Lastly, our analysis leveraged only a small cluster of guaranteed issue states (N = 3) in the sample, which increases the concern that we are detecting significant associations by chance and our estimates are sensitive to influential states that are driving the estimates. Therefore, we conducted a randomization inference,[7] a non-parametric method for assessing the statistical significance of our estimates. In this exercise, we replicate our model n=1,000 times, but in each iteration, we randomly assign the state of diagnosis variable, and generate a distribution of "pseudo-random" estimates (which should cluster around the null). We then calculated a randomization inference P-value, or the probability that a t-statistic would be as extreme or more than the observed t-value of our main estimate (t=8.03). Further, we performed a "leave-one-out" analysis where we sequentially drop all observations from one state and re-estimate our model; this analysis would identify any influential states that may be driving our estimates.

© 2025 Kwon Y et al. *JAMA Health Forum*.

eTable 1. Full stratified analysis results

		Adjusted DD	95% CI	N
**Sex**	Male	2.40***	[1.80,3.01]	444920
	Female	2.65***	[1.89,3.42]	420267
**Race and ethnicity**	Non-Hispanic white	2.63***	[1.86,3.41]	676623
	Non-Hispanic black	1.16***	[0.93,1.40]	65257
	Hispanic	2.58***	[2.18,2.97]	70254
	Other/unknown	3.88***	[2.56,5.20]	53039
**Age**	66-75	3.05***	[2.30,3.80]	383341
	76-85	2.28***	[1.53,3.04]	383068
	86-100	1.45***	[1.05,1.85]	98765
**Stage at diagnosis**	In situ/localized	1.76***	[1.17,2.34]	514190
	Regional	3.35***	[2.72,3.97]	144994
	Distant	4.37***	[3.11,5.63]	161267
	Unstaged	1.70***	[1.04,2.36]	44720
**Cancer type**	Bladder	1.93***	[1.24,2.62]	67212
	Brain	4.82***	[2.65,6.99]	9670
	Breast	2.32***	[1.43,3.21]	174629
	Colorectal	2.11***	[1.58,2.63]	84404
	Kidney	2.13***	[1.53,2.74]	33985
	Leukemia	1.73***	[1.04,2.41]	20068
	Liver	1.67*	[0.19,3.16]	12214
	Lung	3.55***	[2.62,4.47]	111403
	Lymphoma	3.41**	[1.21,5.61]	47899
	Melanoma	0.68***	[0.40,0.97]	94752
	Multiple myeloma	4.03***	[1.94,6.13]	19458
	Ovarian	3.18***	[1.86,4.51]	10548
	Pancreatic	5.15***	[3.73,6.57]	23052
	Prostate	2.19***	[1.56,2.83]	155775
**Yost Index quartile**	Q1	1.70***	[1.21,2.19]	90370
	Q2	3.11***	[2.73,3.49]	121576
	Q3	2.30***	[1.84,2.76]	165533
	Q4	2.67***	[1.87,3.47]	214408
	Q5	2.56***	[1.41,3.71]	252936
**Urbanicity**	Metro	2.43***	[1.78,3.08]	799857
	Non-metro	3.92***	[3.10,4.75]	65336
**Year of diagnosis**	2015	2.90***	[1.90,3.90]	130438
	2016	2.96***	[2.09,3.84]	136583
	2017	2.40***	[2.09,2.70]	152142
	2018	2.27***	[1.66,2.88]	158760
	2019	2.26***	[1.49,3.02]	166822
**MA Plan type**	HMO	2.19***	[1.37,3.01]	566298
	POS	2.36***	[1.90,2.81]	195970
	PFFS	1.92*	[0.20,3.64]	44142
	Local PPO	5.85***	[3.91,7.79]	41070
	Regional PPO	3.89*	[0.27,7.51]	9258
	Other	0.098	[-1.26,1.46]	8309
**Out-of-network coverage**	Yes	3.43***	[2.80,4.06]	244585
	No	2.06***	[1.26,2.86]	620575

© 2025 Kwon Y et al. *JAMA Health Forum*.

**Monthly premium**	Zero-premium	3.25***	[2.45,4.05]	400854
	Q1 (< $20.8)	2.70***	[1.83,3.57]	85148
	Q2 ($20.8 to $43.8)	3.41***	[2.23,4.60]	82928
	Q3 ($43.9 to $74.1)	1.80***	[1.09,2.51]	84080
	Q4 (> $74.1)	1.56**	[0.54,2.59]	84181
**Star ratings**	2 to 2.5	10.3***	[7.57,13.0]	3537
	3 to 3.5	3.95***	[3.41,4.50]	218492
	4 to 4.5	1.68***	[0.91,2.45]	468585
	5	1.74***	[1.47,2.02]	163353
	Missing	1.66	[-6.05,9.36]	11081

Abbreviations: DD, difference-in-differences; CI, confidence-interval; Q, quartile; OON, out-of-network
Notes: Each row plots separate adjusted difference-in-differences coefficient (in percentage-points), estimated among the listed stratification. The model adjusted for county and year fixed effects and beneficiary-level controls, and clustered standard errors at the state-level. This is the full table of Figure 2 in main text.
* P < 0.05; ** P < 0.01; *** P < 0.001

Falsification test results

Among duals, we found small and insignificant changes in switching between states with and without guaranteed issue rights (0.98 pp, $P = 0.183$; eFigure 2),which strengthens confidence that there are no significant time-varying factors that are confounding our main analysis.

eFigure 2. Falsification test using MA beneficiaries who were dually enrolled in Medicaid

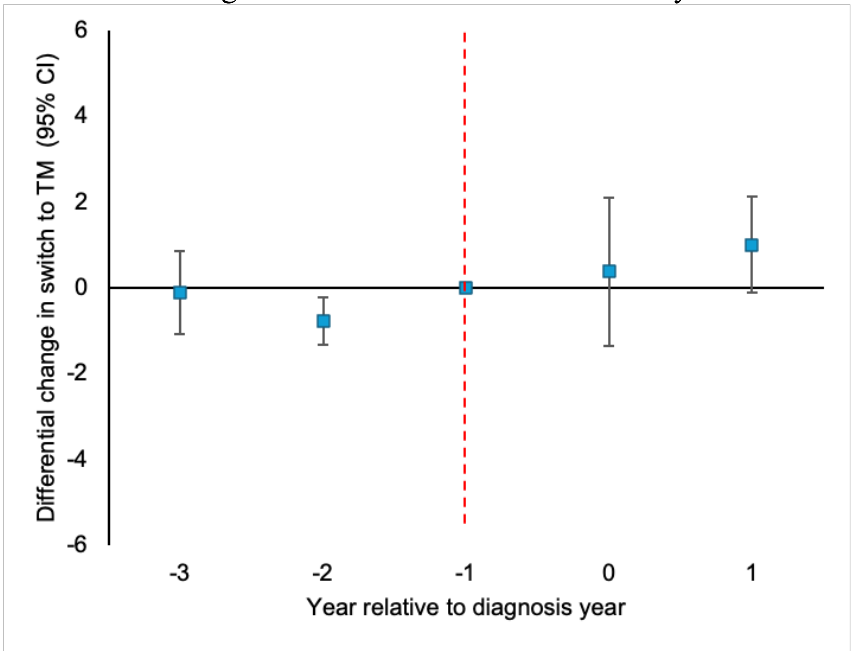

Abbreviations: pp, percentage-points; ci, confidence interval
Note: This plots the event study estimates showing year-specific differential changes in the probability of switching among MA beneficiaries who were dually enrolled in Medicaid *throughout* the enrollment window (i.e., from 4 year prior diagnosis to 1 year after diagnosis) diagnosed in states with and without guaranteed issue rights. The model controlled for county and calendar year fixed effects and clustered standard errors at the state-level. The red dotted line corresponds to the reference period (one year prior to the diagnosis year). The y-axis is in the same scale as that from Figure 1. The pooled coefficient in post-diagnosis period was 0.98 (95% CI: -0.52, 2.48; $P = 0.183$)

© 2025 Kwon Y et al. *JAMA Health Forum*.

Triple difference-in-differences results

eFigure 3 reports on our triple difference-in-differences model, which included beneficiaries without cancer diagnosis (a random 5% sample) as an additional control group. In Panel B, we found that there is a slight differential increase in switching out of TM among the non-cancer cohort in guaranteed issue states, which may reflect overall increasing trends in MA enrollment in this cohort. We difference this out in our main difference-in-differences, finding that MA beneficiaries with cancer had a 1.96 percentage-points higher probability of MA disenrollment (95% CI: 1.42, 2.51; $P < 0.001$) in guaranteed issue states vs. other states, following diagnosis.

eFigure 3. Difference in difference-in-differences between beneficiaries diagnosed with and without cancer

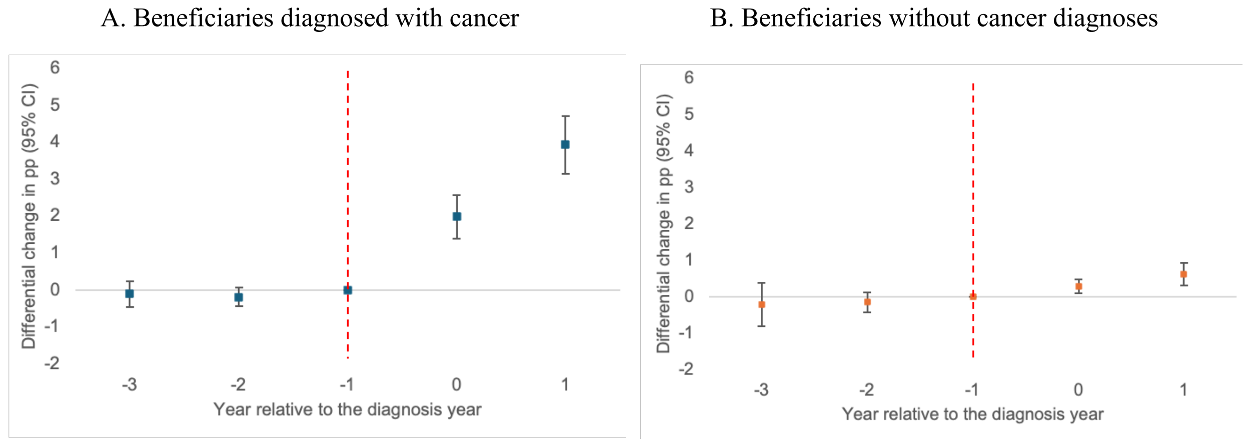

Abbreviations: pp, percentage-points; CI, confidence interval
Note: Exhibit displays event study coefficients examining differential changes in the probability of switching between states with and without guaranteed issue rights among MA beneficiaries with cancer (Panel A) and a 5% random sample of MA beneficiaries without a cancer diagnosis meeting our inclusion criteria. For the non-cancer controls, we randomly assigned a pseudo-date to serve as their index cancer diagnosis date. The model adjusted for county and calendar year fixed effects and clustered standard errors at the state-level. The red dotted line corresponds to the reference period (one year prior to the diagnosis year). The triple difference-in-differences coefficient was 1.96 (95% CI: 1.42, 2.51; $P < 0.001$).

Limiting the sample to the Northeast

When limiting the sample to the SEER states in Northeast, which would improve the balance between exposure and comparison units, we recovered a similar DD estimate as our main specification (DD: 2.0 pp, 95% CI: 1.0, 2.9, *P* = 0.007; eTable 2).

eTable 2. Sensitivity to limiting the sample to the SEER states in the Northeast

	**(1) Main specification**	**(2) Only among Northeastern states**
Difference-in-differences in pp	2.5***	2.0**
95% CI	[1.9,3.2]	[1.0,2.9]
P-value	< 0.001	0.007
Pre-diagnosis mean[a]	2.1	2.1
Relative change[b]	118%	95%
County FE	Yes	Yes
Year FE	Yes	Yes
Beneficiary-level controls	Yes	Yes
N	865,193	232,050

Abbreviations: pp, percentage points; CI, confidence-interval; FE, fixed effects
Note: The exhibit compares the difference-in-differences estimate (and their 95% CI and *P*-values) from our main sample and an alternate sample that only includes observations from states in the Northeast region (where the exposure states were concentrated). The alternate sample only includes New Jersey as the comparison state without any guaranteed issue rights. Both models adjusted for county and year fixed effects and clustered standard errors at the state-level.
[a] Pre-mean is calculated among pre-cancer diagnosis observations in the exposure states with guaranteed issue rights.
[b] Relative change is calculated as the difference-in-differences estimate divided by the pre-diagnosis mean.
** P < 0.05; ** P < 0.01; *** P < 0.001*

Examining differential mortality

In eTable 3, we describe mortality rates between cases diagnosed in states with guaranteed issue rights and other states. The overall 1-year mortality was 26%, but the rate of mortality did not appreciably differ between two set of states (24% vs. 27%; standardized mean difference = 0.049), suggesting that any bias from differential mortality would have been negligible. Indeed, we find that re-estimating our model after including beneficiaries who died in the year of diagnosis (they were excluded in our main sample because we do not observe their plan choice in the year following diagnosis) results in a similar estimate as the main analysis (DD=2.25 pp, 95% CI: 1.60, 2.91, *P* < 0.001).

© 2025 Kwon Y et al. *JAMA Health Forum*.

eTable 3. Mortality rates by states with and without guaranteed issue rights

Mortality proportion	Overall	States with guaranteed issue rights	Comparison states	Standardized mean difference
1-year mortality	0.26	0.24	0.27	0.049
2-year mortality	0.34	0.32	0.34	0.053
3-year mortality	0.38	0.36	0.38	0.052
4-year mortality	0.41	0.39	0.41	0.051
5-year mortality	0.42	0.40	0.43	0.050

Note: This exhibit compares mortality rates between study sample diagnosed in states with guaranteed issue rights in SEER vs. comparison states (all other state registries in SEER).

eTable 4. Sensitivity of main difference-in-differences estimate to including beneficiary-year observations among those who died in the year of cancer diagnosis

	(1) Main specification	(2) Including early decedents
Difference-in-differences in pp	2.53***	2.25***
95% CI	[1.86,3.21]	[1.60,2.91]
P-value	0	0
Pre-diagnosis mean[a]	2.116	2.193
Relative change[b]	120%	103%
County FE	Yes	Yes
Year FE	Yes	Yes
Beneficiary-level controls	Yes	Yes
N	865,193	1,030,311

Abbreviations: pp, percentage points; CI, confidence-interval; FE, fixed effects
Note: The exhibit compares the difference-in-differences estimate (and their 95% CI and *P*-values) from our main sample and an alternate sample added beneficiary-year observations among those who died in the year of diagnosis (i.e., did not live through the next year in which we can observe plan selection). Both models adjusted for county and year fixed effects and clustered standard errors at the state-level.
[a] Pre-mean is calculated among pre-cancer diagnosis observations in the exposure states with guaranteed issue rights.
[b] Relative change is calculated as the difference-in-differences estimate divided by the pre-diagnosis mean.
* P < 0.05; ** P < 0.01; *** P < 0.001

Examining Endogenous mobility

In modeling the probability of any mobility as the outcome, we found a significant but extremely small differential changes among beneficiaries in guaranteed issue states vs. other states (DD: 0.3 pp, 95% CI: 0.08, 0.5, *P* = 0.011; eTable 5). However, endogenous mobility did not impact our main estimate; our estimate was not sensitive to excluding the "movers" from the sample (DD: 2.5 pp, 95% CI: 1.3, 2.1; *P* < 0.001) or using the beneficiary's initial county of residence (DD: 2.4 pp, 95% CI: 1.8, 3.0; *P* < 0.001).

eTable 5. Sensitivity of main difference-in-differences estimate to endogenous mobility

	(1) Mobility as the outcome	(2) Excluding movers	(3) Using initial residence
Difference-in-differences in pp	0.3*	2.5***	2.4***
95% CI	[0.08,0.5]	[1.8,3.1]	[1.8,3.0]
P-value	0.011	< 0.001	< 0.001
Pre-diagnosis mean[a]	0.538	2.2	2.189
Relative change[b]	162%	114%	110%
County FE	Yes	Yes	Yes
Year FE	Yes	Yes	Yes
Beneficiary-level controls	Yes	Yes	Yes
N	865,193	834,149	865,150

Abbreviations: pp, percentage points; CI, confidence-interval; FE, fixed effects
Note: In each column of the exhibit, we show the difference-in-differences coefficients (and their 95% CI and *P*-values) modeling across-state mobility as the outcome (Column 1), estimated after excluding beneficiaries who ever moved across states during the enrollment window (Column 2), and estimated using the beneficiary's *initial* state of residence (from the Medicare enrollment files, rather than SEER files) to categorize exposure and comparison states. All models adjusted for county and year fixed effects and clustered standard errors at the state-level.
[a] Pre-mean is calculated among pre-cancer diagnosis observations in the exposure states with guaranteed issue rights.
[b] Relative change is calculated as the difference-in-differences estimate divided by the pre-diagnosis mean.
** P < 0.05; ** P < 0.01; *** P < 0.001*

Examining covariate trends

As a general check of compositional changes in the sample, covariate trends test was conducted (eTable 6). In this test, we found surprisingly little changes in covariates during the study window, with only 7 covariates showing significant differential changes. However, the size of these estimates is extremely small (less than 1 percentage-points), which helps to rule out compositional changes that may be driving our estimates.

eTable 6. Differential changes in other measured covariates

		Pooled Difference-in-differences in pp		
**Covariate**	**Category**	**Coefficient**	**95% CI**	**P-value**
**Sex**	Male	0.035	[-0.083,0.15]	0.54
	Female	-0.035	[-0.15,0.083]	0.54
**Race and ethnicity**	Non-Hispanic white	-0.14	[-0.36,0.069]	0.172
	Non-Hispanic black	0.12*	[0.029,0.22]	0.014
	Hispanic	-0.0066	[-0.13,0.12]	0.912
	Other/unknown	0.027	[-0.0085,0.062]	0.126
**Age**	65-75	-0.035	[-0.13,0.056]	0.424
	75-85	0.04	[-0.059,0.14]	0.399
	85-100	-0.005	[-0.069,0.059]	0.869
**Stage at diagnosis**	In situ/localized	0.051	[-0.055,0.16]	0.323
	Regional	0.0078	[-0.043,0.058]	0.747
	Distant	-0.075	[-0.16,0.0073]	0.071

© 2025 Kwon Y et al. *JAMA Health Forum*.

	Unstaged	0.017	[-0.016,0.049]	0.288
**Cancer type**	Bladder	-0.012	[-0.050,0.026]	0.516
	Brain and nervous system	-0.0014	[-0.031,0.028]	0.92
	Breast	0.024	[-0.10,0.15]	0.696
	Colorectal	0.025	[-0.011,0.062]	0.159
	Kidney	-0.0048	[-0.043,0.034]	0.793
	Leukemia	0.051**	[0.015,0.086]	0.008
	Liver	-0.027*	[-0.049,-0.0052]	0.018
	Lung	-0.065	[-0.14,0.014]	0.101
	Lymphoma	-0.024	[-0.067,0.020]	0.261
	Melanoma	-0.014	[-0.072,0.043]	0.596
	Multiple Myeloma	-0.033*	[-0.060,-0.0055]	0.022
	Ovarian	-0.0093	[-0.032,0.013]	0.391
	Pancreatic	0.0054	[-0.016,0.027]	0.6
	Prostate	0.085**	[0.025,0.14]	0.008
**Yost Index quartile**	Q1	-0.023	[-0.072,0.026]	0.335
	Q2	0.015	[-0.013,0.042]	0.274
	Q3	-0.04	[-0.085,0.0055]	0.081
	Q4	0.05	[-0.013,0.11]	0.112
	Q5	-0.028	[-0.13,0.072]	0.557
	Missing	0.0076	[-0.0091,0.024]	0.349
**Year of diagnosis**	2014	0.9	[-0.19,1.98]	0.098
	2015	-0.055*	[-0.11,-0.0010]	0.046
	2016	-0.71	[-1.62,0.20]	0.118
	2017	-1.2	[-2.66,0.26]	0.1
	2018	-0.15	[-0.47,0.17]	0.335
	2019	1.22	[-0.37,2.81]	0.124
**Plan type**	HMO	-0.017	[-0.42,0.39]	0.931
	POS	-0.52	[-1.08,0.048]	0.07
	PFFS	0.15	[-0.098,0.40]	0.216
	Local PPO	0.19*	[0.00020,0.38]	0.05
	Regional PPO	0.1	[-0.17,0.38]	0.435
	Other	0.089	[-0.10,0.28]	0.339
**Out-of-network coverage**	Yes	-0.13	[-0.71,0.45]	0.638
	No	0.13	[-0.45,0.71]	0.638
**Monthly premium**	Zero-premium	0.13	[-0.23,0.50]	0.454
	Quartile 1 (< $20.8)	0.049	[-0.20,0.30]	0.683
	Quartile 2 ($20.8 to $43.8)	-0.36	[-0.89,0.17]	0.168
	Quartile 3 ($43.8 to $74.1)	-0.11	[-0.24,0.024]	0.101
	Quartile 4 (> $74.1)	0.12	[-0.13,0.37]	0.324
**Star ratings**	2 to 2.5	0.098	[-0.0047,0.20]	0.06
	3 to 3.5	0.14	[-0.26,0.55]	0.459
	4 to 4.5	-0.25	[-1.02,0.52]	0.505
	5	-0.19	[-1.03,0.65]	0.636
	Missing	0.19	[-0.14,0.53]	0.239

Abbreviations: pp, percentage points; CI, confidence-interval; HMO, Health Maintenance Organizations; PPO, Preferred Provider Organizations; SES, socioeconomic status; POS, Point-of-Service; PFFS, Private fee-for-service; Q, quintile

Note: In each row, we show the difference-in-differences coefficients (and their 95% CI and *P*-values) modeling each level of covariate as the outcome. Each model adjusted for county and year fixed effects and clustered standard errors at the state-level.

* P < 0.05; ** P < 0.01; *** P < 0.001

Randomization Inference Results

In eFigure 4, we plot a histogram of t-statistics, which were generated in each of n=1,000 scenario in which we randomly assign the exposure indicator to the sample. This "pseudo-random" estimates clustered around null, with the mean t-statistic of -0.07. In our test, there was no t-statistics value that was as extreme or larger than that of our main estimate (t=8.03).

eFigure 4. Distribution of pseudo-random estimates from randomization inference test

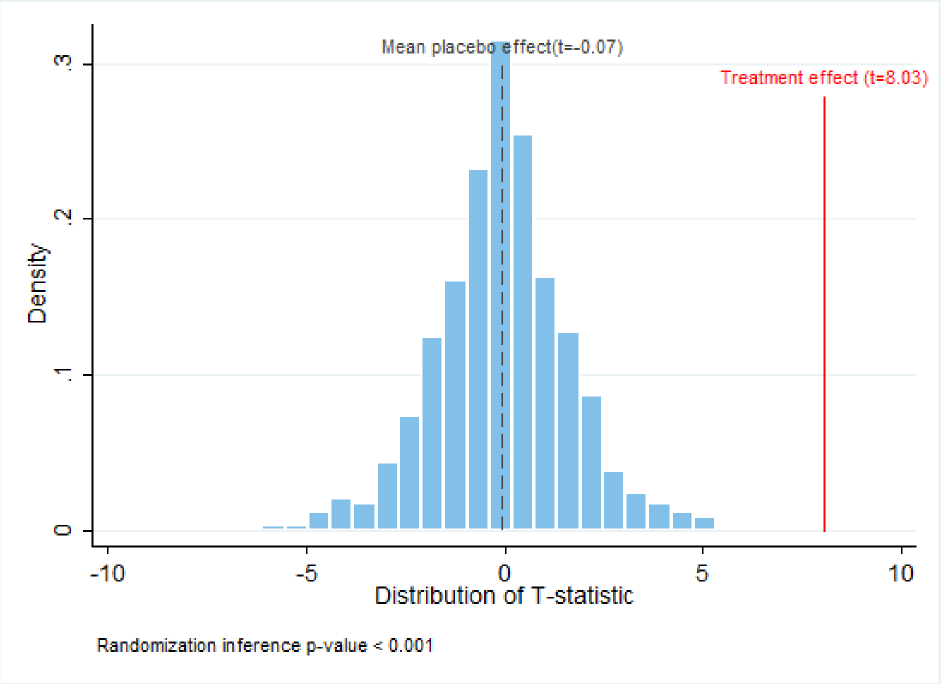

Note: The figure plots the distribution of t-statistics of the pseudo-random difference-in-differences coefficients that were generated in models that randomized the exposure indicator based on the sampling distribution for states in the data (n=1,000 randomizations). We indicate the t-statistic of the mean "placebo" estimate (in gray; t-statistic of the difference-in-difference coefficient = -0.07) and the actual estimate from our main analysis (in red).

Leave-one-out analysis results

In each iteration of leave-one-out analysis, we estimated a DD estimate that was closely similar to our main estimate, with all estimates tightly clustering around 2.53 pp (eFigure 5). We note that excluding New York from the sample substantially increased standard errors (as it constitutes the largest state in the "treated" sample), but still generated a significant estimate at the 5% level.

© 2025 Kwon Y et al. *JAMA Health Forum*.

eFigure 5. Results of "leave-one-out" analylsis

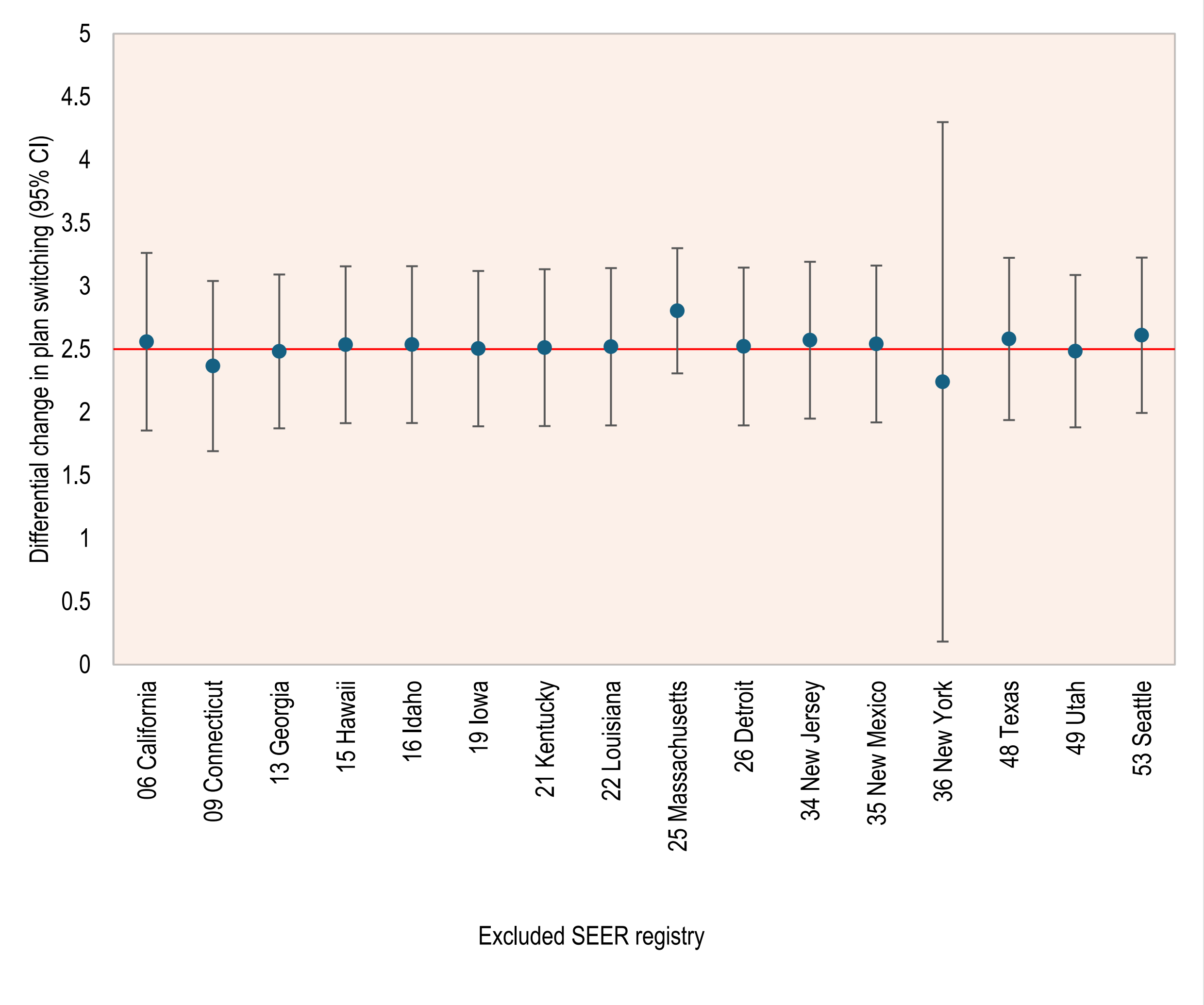

Abbreviations: CI, confidence interval
Note: Each scatter-point corresponds to the difference-in-differences coefficient (and its 95% confidence interval) from each iteration of "leave-one-out" analysis that excluded all observations from the listed state in the *X*-axis. The horizontal red-line equates to the treatment effect from the main analysis (=2.53). All models adjusted for county and calendar year fixed effects and clustered standard errors at the state-level.

© 2025 Kwon Y et al. *JAMA Health Forum*.

eReferences

1. Enewold L, Parsons H, Zhao L, et al. Updated Overview of the SEER-Medicare Data: Enhanced Content and Applications. *J Natl Cancer Inst Monogr*. 2020;2020(55):3-13. doi:10.1093/jncimonographs/lgz029

2. National Cancer Institute. Common Cancer Sites - Cancer Stat Facts. Published online 2024. Accessed December 1, 2024. https://seer.cancer.gov/statfacts/html/common.html

3. Lissenden B. The effect of cancer diagnosis on switching health insurance in medicare. *Health Economics*. 2019;28(3):339-349. doi:10.1002/hec.3843

4. Roberts ET, Glynn A, Cornelio N, et al. Medicaid Coverage "Cliff" Increases Expenses And Decreases Care For Near-Poor Medicare Beneficiaries. *Health Aff (Millwood)*. 2021;40(4):552-561. doi:10.1377/hlthaff.2020.02272

5. Boccuti C, Jacobson G, Orgera K, Published TN. Medigap Enrollment and Consumer Protections Vary Across States. Published online July 11, 2018. Accessed December 1, 2024. https://www.kff.org/medicare/issue-brief/medigap-enrollment-and-consumer-protections-vary-across-states/

6. National Cancer Institute. The SEER-Medicare Data. Accessed January 16, 2025. https://healthcaredelivery.cancer.gov/seermedicare/aboutdata/#

7. Lyu W, Wehby GL. Effects of Virginia's 2019 Medicaid Expansion on Health Insurance Coverage, Access to Care, and Health Status. *Inquiry*. 2022;59:00469580221092856. doi:10.1177/00469580221092856

© 2025 Kwon Y et al. *JAMA Health Forum*.
