# Supplementary material for: Medigap Protection and Plan Switching Among Medicare Advantage Enrollees With Cancer
Source: JAMA Health Forum. 2025 Jun 7;6(6):e252018. doi: 10.1001/jamahealthforum.2025.2018 (PMC12145528; doi:10.1001/jamahealthforum.2025.2018)
Supplement: Supplement 2. — Data Sharing Statement [file jamahealthforum-e252018-s002.pdf]

Data Sharing Statement

Kwon. Medigap Protection and Plan Switching Among Medicare Advantage Enrollees With Cancer. *JAMA Health Forum*. Published June 07, 2025.
doi:10.1001/jamahealthforum.2025.2018

Data
Data available: No

Additional Information
Explanation for why data not available: The datasets used to conduct this study cannot be shared directly as per the research protocol from the National Cancer Institute. Instructions for obtaining these data are available at
https://healthcaredelivery.cancer.gov/seermedicare/obtain/
